# Effects of muscle fatigue on ankle and the fatigue protocols of postural control

**DOI:** 10.1186/1757-1146-7-S1-A111

**Published:** 2014-04-08

**Authors:** YH Shin, CH Youm, YK Kim

**Affiliations:** 1 Sport Biomechanics Laboratory, University of Dong-A, Busan, Korea

## 

Postural control is composed of the integration of vision, vestibular system, and proprioceptive sense along with the balanced control of the musculoskeletal system [1,2]. Muscle fatigue can be defined as the decrease in the maximum muscular strength production capacity due to high intensity or prolonged exercise during physical activity [3,4]. Most studies related to muscle fatigue and postural control use isokinetic dynamometers. Angular speed has mostly been used, being divided into slow [5,6] and fast angular speeds [7,8]. The fast angular speed is mainly used for low intensity, long-duration exercise and the slow angular speed is used for high intensity, short-duration exercise [9,10]. Hence, an understanding of the change in postural control capabilities according to fatigue induction properties of various movements such as plantar flexion and dorsiflexion is deemed necessary.

The purpose of this study was to investigate the effects of muscle fatigue on ankle joint and the fatigue protocols of postural control during single-leg stance. The subjects of this study were 24 healthy adult women. Fatigue was induced on plantar flexion and dorsiflexion with an isokinetic dynamometer at angular velocities of 30 °/s and 120 °/s.

Among the anteroposterior plane factors, plantar and dorsiflexion resulted in decreased postural control during single-leg stance after fatigue induction using a plantar and dorsiflexion fatigue protocol at an angular velocity of 30 °/s. No change was observed in the postural control during single-leg stance postural control on application of the fatigue protocol at an angular velocity of 120 °/s. Plantar and dorsiflexion did not differ significantly with the application of the fatigue protocol at angular velocities of 30 °/s and 120 °/s. Among the mediolateral plane factors, postural control diminished during single-leg stance after fatigue induction on application of the plantar and dorsiflexion fatigue protocol at an angular velocity of 30 °/s. On application of the fatigue protocol an angular velocity of 120 °/s, however, no change in the postural control was observed during single-leg stance. Thus, the plantar and dorsiflexion fatigue protocol applied at an angular velocity of 30 °/s resulted in decreased single-leg stance postural control compared to that observed at an angular velocity of 120 °/s.

In summary, during high-intensity, short-duration exercise involving plantar and dorsiflexion at an angular velocity of 30 °/s, fatigue at 50% of the maximum plantar flexion torque might result in reduced single-leg stance postural control.
